# An Unsupervised Method for Artefact Removal in EEG Signals

**DOI:** 10.3390/s19102302

**Published:** 2019-05-18

**Authors:** Angel Mur, Raquel Dormido, Natividad Duro

**Affiliations:** Department of Computer Sciences and Automatic Control, Universidad Nacional de Educación a Distancia (UNED), Juan del Rosal 16, 28040 Madrid, Spain; raquel@dia.uned.es (R.D.); nduro@dia.uned.es (N.D.)

**Keywords:** artefacts, EEG, artefact detection, artefact removal, ICA

## Abstract

Objective: The activity of the brain can be recorded by means of an electroencephalogram (EEG). An EEG is a multichannel signal related to brain activity. However, EEG presents a wide variety of undesired artefacts. Removal of these artefacts is often done using blind source separation methods (BSS) and mainly those based on Independent Component Analysis (ICA). ICA-based methods are well-accepted in the literature for filtering artefacts and have proved to be satisfactory in most scenarios of interest. Our goal is to develop a generic and unsupervised ICA-based algorithm for EEG artefacts removal. Approach: The proposed algorithm makes use of a new unsupervised artefact detection, ICA and a statistical criterion to automatically select the artefact related independent components (ICs) requiring no human intervention. The algorithm is evaluated using both simulated and real EEG data with artefacts (SEEG and AEEG). A comparison between the proposed unsupervised selection of ICs related to the artefact and other supervised selection is also presented. Main results: A new unsupervised ICA-based algorithm to filter artefacts, where ICs related to each artefact are automatically selected. It can be used in online applications, it preserves most of the original information among the artefacts and removes different types of artefacts. Significance: ICA-based methods for filtering artefacts prevail in the literature. The work in this article is important insofar as it addresses the problem of automatic selection of ICs in ICA-based methods. The selection is unsupervised, avoiding the manual ICs selection or a learning process involved in other methods. Our method is a generic algorithm that allows removing EEG artefacts of various types and, unlike some ICA-based algorithms, it retains most of the original information among the artefacts. Within the algorithm, the artefact detection method implemented does not require human intervention either.

## 1. Introduction

The activity of the brain can be recorded by means of electroencephalogram (EEG). EEG is a noninvasive method that uses a set of sensors (electrodes) distributed along the scalp. The artefacts are signals recorded by the EEG with no connection to a particular brain activity. Artefacts can be of physiological or nonphysiological origin. Physiological artefacts are generated by the patient (e.g., ocular movements, eye blinks and muscular activity) and nonphysiological artefacts can arise from outside the body (e.g., equipment, environment).

Artefacts can be marked or detected. Once the artefacts are known, they can be directly removed by rejecting the segments of the signal containing the artefacts. In this case, there could be a considerable loss of useful information from the EEG signal. Rejecting contaminated EEG segments can be applied when segments contain excessive interference [1,2], but, in general, it is desirable to filter only the artefacts while retaining as much information of the EEG signal as possible [3]. This filtering without rejecting the cognitive part is important in the brain-computer interfaces and short recordings [4,5].

Urigüen et al. [1] provides a detailed review about EEG artefact removal methods. There are different approaches such as regression [6], ocular artefact correction [7], filtering [8] or BSS techniques [9,10]. In general, the BSS methods are commonly used and mainly those based on ICA [9,11]. These methods take advantage of the independence between the artefacts and the brain activity.

The BSS methods can also be classified as semiautomated or automated. Semiautomated methods require visual inspection by experts either to detect artefacts or to classify the resulting components as artefacts components or cognitive components. This strategy applies for offline applications and, in general, it is a tedious procedure. Furthermore, the criterion used by the expert could not be uniform during the analysis. To avoid these problems, an automatic procedure is preferred. However, an automatic artefact elimination process is not a trivial problem since artefacts are mixed with each other and with the EEG signal in very different ways.

The first step to remove an artefact is to detect it. There are different approaches for detecting artefacts. For example, statistical methods and/or threshold values can be used [2,12,13,14]. Classification methods are an alternative to detect artefacts, for example, DETECT [15] and the unsupervised method for event detection (UMED) [16]. These methods detect artefacts by means of an identification or characterization procedure.

Once an artefact has been detected, an ICA can be applied to the EEG signal to obtain a set of ICs. Some of these components are related to the brain activity and the rest to the artefact. A method must divide the components into those two groups. Then, the components of the brain activity are used to rebuild a corrected EEG signal by eliminating the contributions of the artefactual sources.

When an EEG portion presents an artefact, the cognitive part whose origin is in the brain is considered independent of the artefact whose origin is outside the brain. On the other hand, in the ICA algorithms, the number of desired ICs is an input argument. When a number of 2 is selected, the result, in most cases, is a poor approximation of the cognitive part and the artefact. Therefore, to obtain an acceptable result, it is better to work with a higher number of ICs.

The EEG signal is captured using a set of *N* electrodes. The ICA methods offer a maximum number of ICs equal to the number of electrodes. Since, in general, the optimum number of ICs is greater than *N*, it is necessary to work with the highest possible number of components (that is *N*). However, when the number of components increases, the problem of how to automatically group the cognitive components and the components related to the artefact arises.

MARA [17] is a technique built on ICA that allows users to automatically determine those components of ICA that do not come from brain activity. For this purpose, MARA uses a classifier (a Linear Programming Machine) that uses some features that allow discrimination of an ICA component whose origin is not in brain activity. It can handle any type of artefact.

The SASICA software [18] provides a practical guide to select artefactual ICs of EEG. Normally, this selection needs to be improved analysing each IC. Consequently, this filtering process is semiautomatic.

wICA [19] is an ICA-based method for filtering artefacts that includes a wavelet threshold of ICs as an intermediate step. This filtering is automatic, but the result depends on some parameters to determine the wavelet threshold.

There are other recent research papers that present artefact denoising of EEG signals. Melia et al. [20] presents an algorithm for removing peak and spike noise from EEG. It is based on filtering and thresholding the analytic signal envelope. Mahajan et al. [21] presents an unsupervised algorithm that uses modified multiscale sample entropy and Kurtosis to automatically identify the independent eye blink artefactual components and, subsequently, denoise these components using biorthogonal wavelet decomposition. This method does not require manual identification for artefactual components. The method FORCe [22] is an artefact removal method developed for use in brain-computer interfacing. It is based on a combination of wavelet decomposition, ICA and thresholding. The EEMD-ICA approach [23] removes artefacts, preserving more information than other classical ICA methods. Chen et al. [24] proposes to filter both ocular and muscle artefacts by exploiting diverse statistics. Somers et al. [25] presents a generic algorithm based on the multichannel Wiener filter for removal of EEG artefacts of various types.

Mannan et al. [26] and KafiulIslam et al. [27] provide a comparative study of various EEG artefact removal techniques. Mannan et al. [26] also review the schemes developed for validating the performances of algorithms with simulated and real EEG data.

The main goal of this paper is to present a generic and unsupervised ICA-based method to automatically remove artefacts (UAR). The method combines a new unsupervised artefact detection algorithm, ICA and a new unsupervised algorithm to select ICs related (or not) to brain activity. The UAR offers a solution to the problem of automatic selection of ICs in ICA-based method using a statistical criterion to select the ICs for artefact removal while preserving most of the original information between artefacts. It is not limited to a specific number or type of artefact and it can also be applied in online applications.

The performance evaluation of artefact removal methods is problematic due to the lack of standard quantitative metrics in the literature to measure both the amount of artefact removal and the distortion [1,26,27]. Most of the methods are often tested on real EEG, and their performances are evaluated in terms of some qualitative plots analysed by visual inspection. UAR results will be evaluated by visual inspection on AEEG and SEEG. A metric will also be used to evaluate the SEEG filtering. Comparisons are also presented between UAR, MARA, SASICA and wICA.

There is a large number of algorithms developed for artefact removal from EEG signals. However, there is no universal solution available yet and, in general, it is not fair to say which method performs best. The filtering quality of a particular method varies according to the conditions of use (type of artefacts, number of electrodes, etc.). For example, an ICA-based method can filter electromyographic (EMG) artefacts worse than other methods when the number of electrodes is small, but can obtain a better performance with a high number of electrodes. Regarding the number of electrodes, Chen et al. [28] proposes to use single channel techniques for the removal of muscle artefacts instead of using multichannel approaches. This study used a maximum of 23 channels.

For these reasons, in this paper it is shown that the quality of the proposed UAR filtering is at least as good as that of other methods (in our case comparing UAR with MARA, SASICA and wICA).

The paper is organized as follows. Section 2 presents some background necessary to implement the UAR. Section 3 describes the new artefact detection method used in UAR. The proposed method to filter artefacts in EEG recordings is presented in Section 4. In Section 5, the method is tested using some EEG recordings (both AEEG and SEEG) with different types of artefacts. Finally, in Section 6 and Section 7, a discussion and conclusions of the paper are presented, respectively.

## 2. Background

This section briefly explains a method to detect events (UMED), the ICA algorithm and the two-sample variance test.

### 2.1. Unsupervised Method for Event Detection

The Unsupervised Method for Event Detection (UMED) method [16] is used to detect events in a multichannel signal in an unsupervised way. In this method, the multichannel signal is divided into a set of intervals created from a sliding window (with a sliding distance of d samples). Each interval is represented by a vector of autoregressive coefficients AR (2) (two for each channel) [29].

Using several windows, the hierarchical algorithm [30] and the S_Dbw index [31], the optimal window *L_w_* that classifies the intervals in an unsupervised way is found. Those consecutive intervals belonging to the same group form a state. The transition between two states represents an event.

UMED performs an unsupervised classification to detect events. In this paper, a new method for artefact detection that avoids this unsupervised classification is presented in Section 3. The implemented method makes use of the optimal window *L_w_* and the autoregressive coefficients AR defined in UMED. AR coefficients are reliable feature sets to detect artefacts in EEG recordings [32].

The UMED method is also part of the unsupervised method for event detection and classification of multichannel signals (UMEDC) [33].

### 2.2. ICA

Given a set of *n* mixed signals formed by combining *k* independent signals with *p* samples, its ICA model is defined as M=A×S, where *M* is a n×p data matrix, *A* is a n×k mixing matrix and *S* is a k×p matrix of independent signals.

The objective of ICA is to calculate *A* and *S* knowing only *M*. ICA does not need any knowledge concerning the nature of the source signals or their proportions. To estimate *A*, ICA requires the pure signals in *S* to be truly independent and non-Gaussian. Both conditions are usually met when the sources are real signals.

The independence in ICA can be reached by maximizing the non-Gaussianity of the components or by minimizing the mutual information [34]. Around this concept, different ICA algorithms have been developed: FastICA [35], JADE [36], InfoMax [37], Stochastic Non-Negative Independent Components Analysis [38], RADICAL [39], etc. For EEG artefact removal, SOBI, InfoMax and FastICA are mainly used [40,41].

The FastICA algorithm outperforms most of the commonly used ICA algorithms in convergence speed. Such property becomes useful in this article since ICA is applied individually for each artefact. FastICA decompositions are somewhat unstable (for the same signal, the ICs are not always reproduced in the same way). However, this fact is not a problem for the proposed UAR algorithm.

The FastICA algorithm applied to an artefact requires a minimal window length so that some ICs can capture characteristics of the artefact. In general, a window length above 10 s could be enough. In this paper we will use a window length of 26 s (see explanations in Section 5.3).

Given an EEG signal of *N* electrodes (*E*_1_, …, *E_N_*), the FastICA outputs a set of ICs (*CO*_1_, …, *CO_N_*) and its corresponding mixing matrix *A*. For a specific independent component, *CO*, we denote its projection over an electrode *E* as *Projection* (*E*,*CO*). If the signal of the electrode *E* is *M_E_*, then

(1) $$M_{E}=\underset{i}{\sum }Projection\left(E,CO_{i}\right)$$

### 2.3. Two-Sample F-Test for Equal Variances

The two-sample *F*-Test [42] is used to test if the variances of two populations (*X*1 and *X*2) are equal. The test statistic is a ratio of the two sample variances (*v*1 and *v*2). This ratio is called the *F*-statistic and follows an *F* distribution: *F* = *v*1/*v*2. The null hypothesis *H*0 assumes that the variances are equal and the ratio is one. The alternative hypothesis *H*1 assumes that *v*1 and *v*2 are different, and that the ratio deviates from unity. *H*0 is rejected if *F* is either too large or too small. The test uses a significance level (normally at the 5% or 1%) when rejecting *H*0. This test is based on two assumptions: (a) the samples are normally distributed and (b) the samples are independent of each other.

In this paper, *R*1 denotes when the two-sample *F*-Test rejects the null hypothesis at the 1% significance level and *R*0 otherwise.

## 3. Method for Artefact Detection Used in UAR

In this section, we present a new artefact detection method (AD_UAR). It has been developed based on our previous UMED algorithm. An AEEG is used to show how it works.

### 3.1. EEG Data, UMED and the Artefact Detection Method

**Figure 1:**
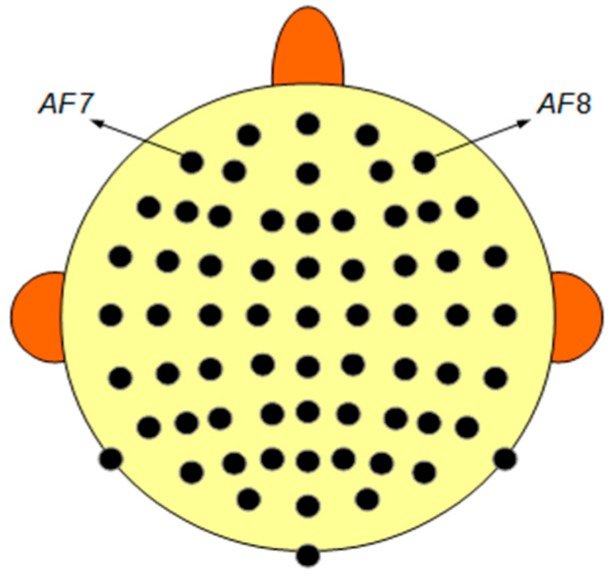
Distribution of the electrodes on the scalp. AF7 and AF8 are the electrodes of the signals used in the graphical representations.

In this paper, the test signal is an 8-min EEG recording used in [15,16] to show the performance of DETECT toolbox and the UMED method. The data are sampled at 256 Hz using a 64-channel Biosemi Active Two System. All 64 channels are used. This signal presents different kinds of physiological artefacts: Jaw Clench, Jaw Movement, Eye Blink, Eye Left Movement and Eye Up Movement. Figure 1 represents the distribution of the electrodes on the scalp. The signals of the AF7 and AF8 electrodes will be used in the graphical representations in this paper.

**Figure 2:**
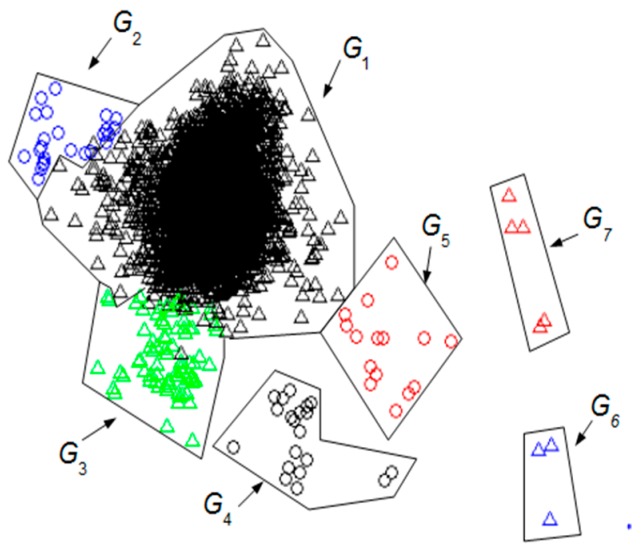
Groups of intervals found using unsupervised method for event detection (UMED) and displayed by means of a PCA. The *L_w_* has 155 samples. The first two principal components contain 55% of the full information.

In Section 2.1 UMED’s basic principles have been presented (full details can be found in [16]). Using UMED on the AEEG described above (*L_w_* = 155 samples and *d* = 32 samples) some clusters of intervals are found. As Figure 2 shows, these groups can be represented by means of a Principal Component Analysis (PCA) [43]. Each cluster can be distinguished by a specific shape and colour (full details about Figure 2 can be found in [16]).

The most numerous group *G*_1_ consists of intervals that represent the cognitive activity of the brain without artefacts. The other groups correspond to different types of artefacts: winks, face movements, etc. As we will analyse later, you cannot rule out finding some intervals in *G*_1_ with a small artefact activity.

The new AD_UAR is based on an analysis of the distances (for example, Euclidean distance) between all the intervals and their centroid. A histogram is used to carry out this analysis.

**Figure 3:**
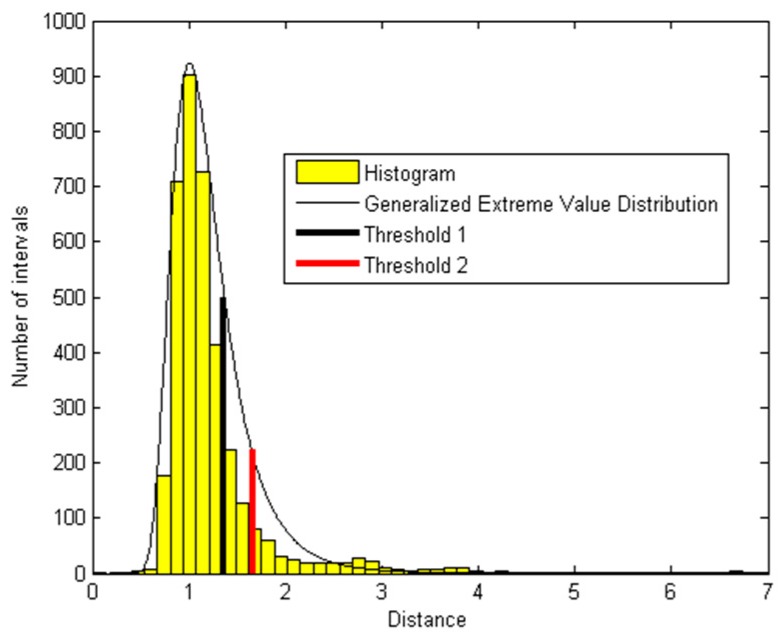
Histogram of interval distances and its generalized extreme value distribution.

Figure 3 shows the histogram that can be modelled by a Generalized Extreme Value Distribution. We consider two thresholds: *T*_1_ and *T*_2_. *T*_1_ points at the beginning of the extreme tail of the histogram. *T*_2_ value is chosen so that beyond *T*_2_ the number of intervals is the same as within [*T*_1_, *T*_2_]. All the intervals containing artefacts (included from *G*_2_ to *G*_7_) are beyond *T*_2_. Some intervals with a low artefact activity can be found next to *T*_2_. For our test signal the value of *T*_1_ is 1.33 and of *T*_2_ is 1.66.

**Figure 4:**
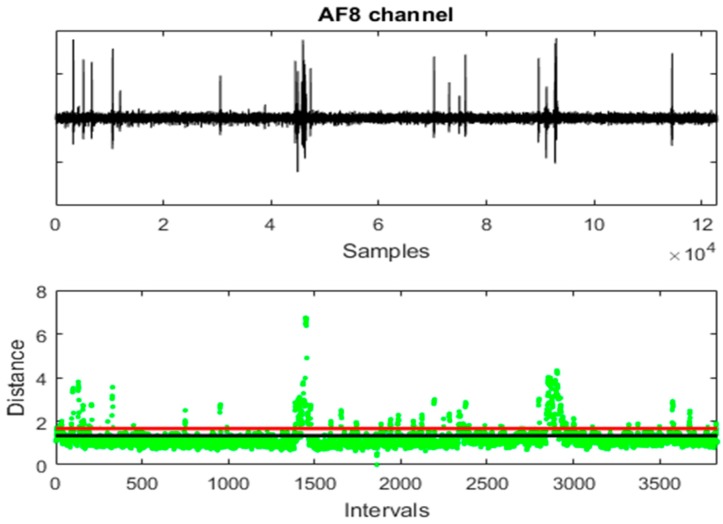
An EEG channel, the distances of the intervals to their centroid and the thresholds *T*_1_ (in black) and *T*_2_ (in red).

Figure 4 shows an EEG channel, the distances of the intervals to their centroid and the thresholds *T*_1_ and *T*_2_. All the channels have been used to calculate the distances. It is simple to infer that the distance between the intervals and their centroid is a good quantitative measure to detect artefacts.

Given a specific threshold *T* (for example *T* = *T*_1_), any piece of the signal formed by consecutive intervals with a distance greater or equal to *T* is a potential portion of the signal containing artefacts.

The artefact detection method (AD_UAR) can be summarized as follows.

Given an EEG signal of *N* channels, a sliding window *L_w_*, a sliding distance *d*, a number of channels (or electrodes) *n* (1 <= *n*<= *N*) and a threshold *T*:

(1) Calculate the intervals (and their autoregressive vectors) of the AEEG using *L_w_*, *d* and *n*.

(2) Calculate the distances between the intervals and their centroid.

(3) Determine potential portions of signal containing artefacts by selecting consecutive intervals with a distance greater or equal to *T*.

### 3.2. Selection of d, T and L_w_

The sliding distance *d* has to be small, but it is not necessary to choose *d* = 1 to obtain a good detection. In this paper we have chosen *d* = 32 samples.

To set the value of *T* let us think about some possible situations that could happen in the detection process.

The artefacts can be mixed in a different way for each channel. This means the artefact recorded at each electrode varies (it can be stronger or weaker) depending on the electrode position. When the number of electrodes *n* is high and the portion of the signal containing the artefact is not significantly different from the signal without artefact (on most electrodes), the AD_UAR could not always be precise enough to detect artefacts.

**Figure 5:**
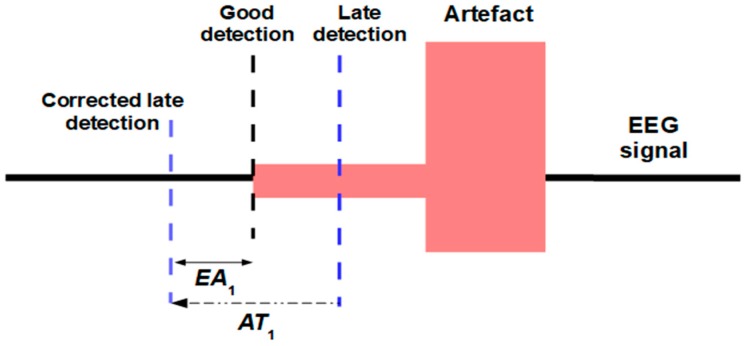
An artefact and two types of detection.

Figure 5 shows two types of detection at the beginning of an artefact. When the artefact is well represented in most of the channels, the AD_UAR offers a “good detection”. There is a “late detection” when the artefact weakly changes the AEEG. For example, if the beginning of the artefact is weak. A similar phenomenon can also appear at the end of the artefact with an “early detection”.

To avoid these possible detection problems the following solutions are proposed.

- Option A (*OA*): Select in AD_UAR a threshold of *T*= *T*_1_. That is, *T* is set to the threshold where the tail of the Generalized Extreme Value Distribution begins.
- Option B (*OB*): Increase the limits of the detected artefact portion.

*OA* tries to find all the potential artefacts. Any threshold between *T*_1_ and *T*_2_ could be a candidate for *T*. However, as it is not possible to know a priori which is the best one for a signal, threshold *T* = *T*_1_ is chosen. In this way, portions of signal with distances between *T*_1_ and *T*_2_ will be analysed using UAR. Although it is expected that most of these portions of signals will not need to be filtered, it is guaranteed that some intervals with a potential artefact will not be lost. However, this will imply more processing.

Threshold *T*_1_ = 1.33 should not vary significantly with another signal. Thus, this value could be chosen for another AEEG with 64 electrodes without the need to analyse a new Generalized Extreme Value Distribution. This is explained by the following paragraph of Ref. [16]. “The artefacts are very well-differentiated from the cognitive EEG part without artefacts. This cognitive part is only related to the thinking and its high variability is concentrated in a unique cluster that is the biggest. This cluster is not affected using different subjects since the AR coefficients are scale- and location-invariant.”

If the number of channels *n* is high, *OA* could not be enough to correctly detect the beginning and the end of some artefacts. This is so since the Euclidean distance is less sensitive to small changes. This problem is solved using *OB.*

With *OB*, all the potential artefacts detected are widened. For example, in Figure 5 the “late detection” is expanded to the left by means of the *AT*_1_ portion. If the *AT*_1_ size is appropriate, the possible “late detections” are corrected. In general, for any type of detection, a small part of the signal without artefact will be added to the detected artefact portion. In Figure 5, the “corrected late detection” adds the *EA*_1_ portion to the left of the artefact. Analogously, it is understood that the detected end of the artefact expands to the right in order to correct “early detections”.

It is not possible to automatically know how good the detection is, and consequently all the potential artefacts detected are widened in the same way. The UAR algorithm will filter each artefact using the new limits established by *OB*. The UAR filtering will hardly modify the «*EA*» portions.

When the number of channels is high and the artefact appears in one electrode or in a very small number of electrodes, the AD_UAR algorithm (using all the channels) may not detect it, even using a *T* = *T*_1_ for the *OA* explained before. In this case, it is better to use AD_UAR for each channel individually (or in small groups of channels) and then fuse the results of all the channels (or groups). In this paper, we have preferred to test the UAR algorithm using all the channels for the AD_UAR algorithm.

Finally, the choice of *L_w_* = 155 is motivated by the UMED result. With another AEEG, *L_w_* can be different. However, if UMED is not used to determine *L_w_*, the window *L_w_* = 155 could also give good filtering results since *OB* corrects the lack of detection accuracy. The accuracy of the detection can decrease if the window is not optimal, for example, to detect consecutive artefacts. In our article, accuracy is not decisive because consecutive intervals are considered as a single artefact and also both the beginning and the end of an artefact are extended by *OB*.

Consequently, in this paper, we will work with *d* = 32, *n* = 64, *L_w_* = 155 and *T* = *T*_1_ = 1.33. The selection of the increased limits for *OB* will be specified in the next section.

## 4. Unsupervised Method for Artefact Removal

This section explains the new Unsupervised Method for Artefact Removal (UAR) proposed in this paper.

### 4.1. Previous Notation

**Figure 6:**
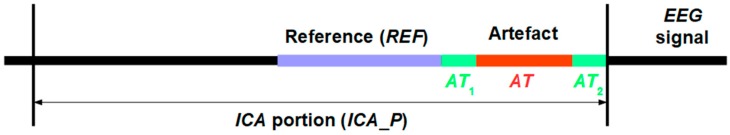
Notation of some parts around a detected artefact (for an EEG channel) used in the automatically remove artefacts (UAR) algorithm.

Figure 6 shows some pieces around a detected artefact (*AT*) used in the UAR algorithm. When an artefact is detected using the AD_UAR algorithm, *OB* adds two portions of the signal around *AT* (*AT*_1_ and *AT*_2_). The UAR algorithm will filter the portion *ART* = *AT*_1_ + *AT* + *AT*_2_. *ICA*_*P* is the portion of the signal where the ICA (FastICA) performs. The reference (*REF*) is a reference portion used by UAR to carry out the two-sample *F*-Test (see Section 2.3).

In this paper, the duration of *ICA_P* is 26 s (see explanations in Section 5.3), *AT_1_ = AT_2_* = 1 s and the length of *REF* is equal to the length of *ART*. If a new artefact starts inside *AT_2_*, then *AT_2_* is selected to a point less than or equal to the beginning of this new artefact. The *AT_1_* of the new artefact will start at that selected point.

### 4.2. The UAR Method

Once an artefact has been detected using the AD_UAR algorithm the basic idea of the UAR method is to calculate an ICs set by selecting a portion of signal according to the schema of Figure 6. Then, the ICs are arranged in descending order from the one that most influences the artefact to the less. Finally, following the ordered ICs found, the filtering process subtracts iteratively, for each electrode and only in the *ART* part, the projection of each IC. The two-sample F-Test (see Section 2.3) allows comparing each subtraction with *REF* and stopping the iterative process.

The UAR method can be summarized as follows.

Given an AEEG of *N* electrodes:

Step 1: Artefact Detection. The potential artefacts are detected by means of the *AD*_*UAR* method.

Step 2: ICs. For each artefact and according to the notation in Section 4.1, the FastICA algorithm is applied to obtain the *N* ICs. The ICs and ICA_P have the same length.

Step 3: ICs in order. Each IC is projected on all the electrodes. The projection of the electrode with, for example, the maximum absolute value (or the maximum variance) in the artefact portion, is selected. Then, the ICs are sorted in a descending way comparing the selected maxima. The ordered ICs are denoted with: *C_1_, …, C_N_.*

Step 4: Filtering process. For each electrode in the set (*E*_1_, …, *E_N_*), the variance of *REF* is compared with the variance in *ART* each time the projection of a *C_i_* is filtered starting in *C*_1_.

For example, with the electrode *E* (see Algorithm 1).


**Algorithm 1. The filtering process with the electrode E**
  *N_ART* ← *ART*; *C* ← *C*_1_; *STOP* ← *0; FIRST* ← 0; *BEFORE* ← 0; *i* ← 1;  while *STOP* ← *0*    *N*_*ART* ← *N*_*ART* - Projection *(E, C, ART) //Filtering process*    if variance(*REF*) > variance(*N*_*ART*) and *R*1     *STOP* ← 1 and *ART* ← *N_ART*     if *BEFORE* ← 1         *ART* ← *OLD*_*ART*     end    end    if *R*0     if *FIRST*← 0         *OLD*_*ART* ← *N_ART*     end     *FIRST* ← 1 and *BEFORE* ← 1    else     *FIRST* ← 0 and *BEFORE* ← 0    end    *i* ← *i* + 1; *C* ← *C_i_*  end

Explanation: The filtering process for an artefact and an electrode *E* consists in subtracting the projection of each independent component following the ordered set of ICs (*C*_1_, …, *C*_N_). The *Projection (E*, *C*, *ART*) denotes the selection of the “*ART* portion” from the projection of *C* over *E*. In each iteration this subtraction is saved in *N*_*ART*. The algorithm stops (*STOP* ← *0*) when the variance of reference *REF* is bigger than the variance of *N*_*ART* and the two-sample *F*-Test (between *R* and *N_ART*) rejects the null hypothesis at the 1% significance level (meaning the result of the test is *R*1). In this case, the *ART* portion is replaced by the result of the final subtraction (*ART* ← *N_ART*). If previously there is a consecutive sequence of *R*0 (*BEFORE* ← 1), then the *ART* portion is replaced by the first portion (*FIRST* ← 0) of that sequence (*ART* ← *OLD_ART*).

The UAR algorithm works each artefact individually respecting the AD_UAR detection order. Each detected portion may represent one or some consecutive artefacts.

## 5. Testing the *UAR* Algorithm

In this section, we filter the AEEG described in Section 3.1 using the UAR algorithm. Then, using that AEEG, we compare UAR with MARA, SASICA and wICA. We also use a SEEG that has been created by mixing a real EEG signal without artifacts (REEG) and some simulated artifacts. Finally, using that SEEG signal we compare UAR with MARA, SASICA and wICA.

### 5.1. Filtering an AEEG Using UAR

The parameters used for the AD_UAR method are *d* = 32; *n* = 64; *L_w_* = 155; and *T* = *T*_1_ = 1.33 for the AD_UAR method, and for the rest of the UAR algorithm: length{*ICA*_*P*} = 26 s; length{*AT*_1_} = length{*AT*_2_} = 1 s; length{*REF*} = length{*ART*}; and 1% significance level for the two-sample F-Test.

**Figure 7:**
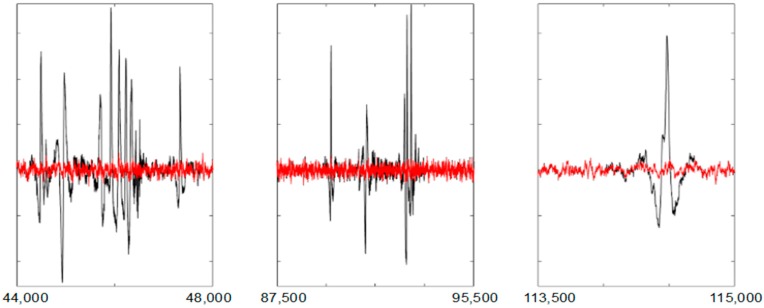
Three sequences of artefacts in the AF8 channel. In red, the filtered signal. The *X* axes show the samples.

Figure 7 shows three artefacts sequences on the AF8 channel. They have been filtered with *UAR*.

**Figure 8:**
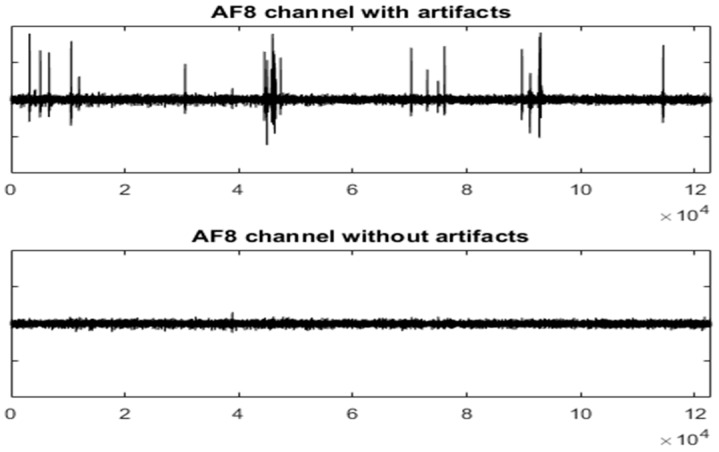
The AF8 channel of AEEG with and without artefacts. The *X* axes show the samples.

**Figure 9:**
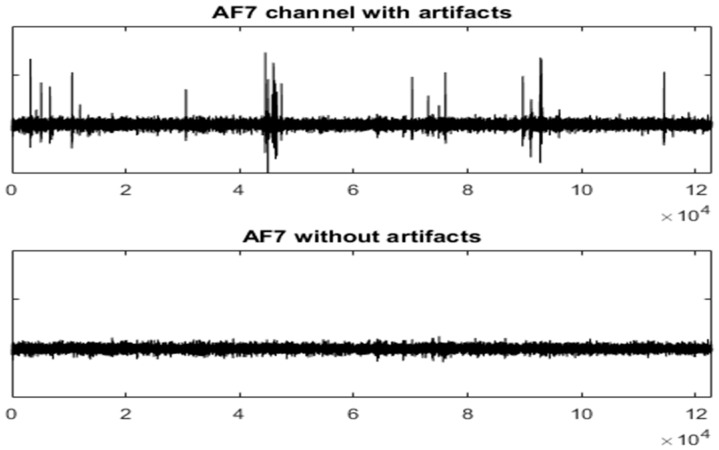
The AF7 channel of AEEG with and without artefacts. The *X* axes shows the samples.

Figure 8 and Figure 9 show the entire channels AF8 and AF7 without and with filtering.

**Figure 10:**
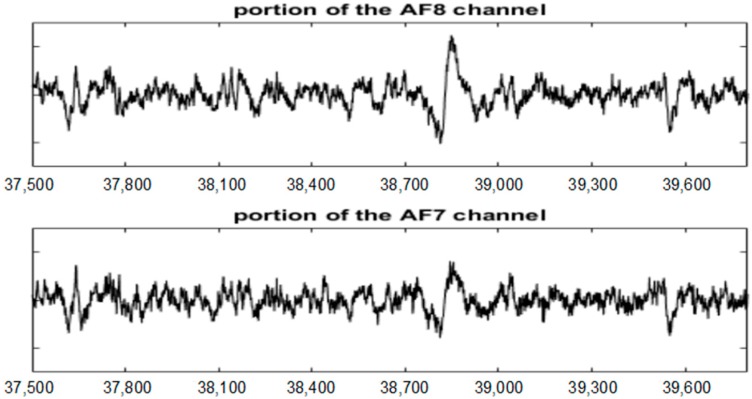
The undetected artefact in the EEG portion [37,500 39,800] of the channels AF8 and AF7.

As displayed in Figure 8, after filtering in the AF8 channel, a small artefact appears near the sample 40,000. This artefact has not been filtered because it has not been detected. The reasons for this are threefold: the artefact is small, it appears in a very small number of channels and the number of electrodes is high. The AF7 electrode is not far from AF8; this artefact has lost its importance in relation to the rest of the signal. This artefact has a maximum amplitude in the area of AF8 and is losing strength when we move away from that area (see Figure 10).

The figures show the quality of the UAR performance.

### 5.2. Filtering an AEEG Using MARA

The EEGLAB is an interactive toolbox for processing electrophysiological data incorporating ICA and artefact rejection. This toolbox presents some extensions for artefact removal, for example, the MARA extension [17]. MARA is a linear classifier that learns from expert ratings by extracting six features from the spatial, the spectral and the temporal domains. This classifier and UAR pursue the same objectives. However, MARA is a supervised method.

In this section, we compare UAR with MARA using the AEEG described in Section 3.1. Instead of creating a new metric, both methods will be visually compared.

**Figure 11:**
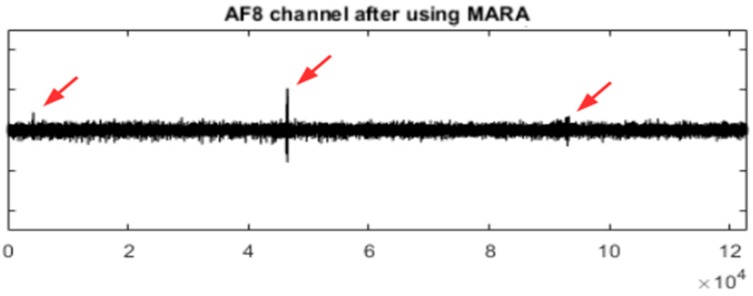
The AF8 channel of AEEG after using the MARA method. The red arrows point to some artefacts that have not been well filtered.

Figure 11 shows the EEG signal in the AF8 channel after using the MARA method. When comparing Figure 8 and Figure 11, it is observed that UAR performs better for some important artefacts (see the red arrows). However, MARA is able to filter the small artefact near sample 40,000.

**Figure 12:**
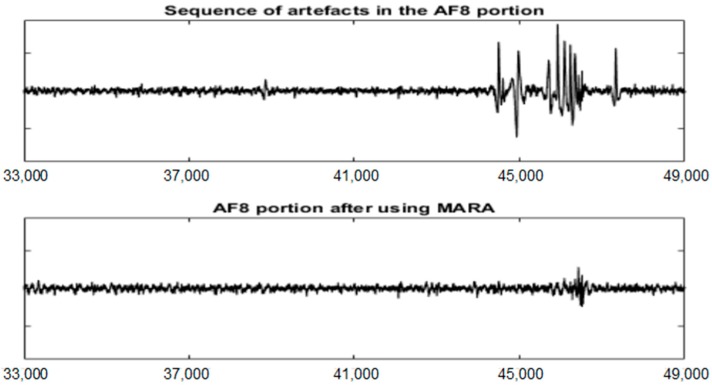
The EEG portion [33,000 49,000] of the AF8 channel before and after using the MARA method.

The quality of the MARA result depends on its training process but it is also important the way in which the ICs are obtained. Figure 12 shows the MARA result after filtering only the EEG portion in the interval [33,000 49,000]. In this case, the filtering seems to be better although an artefact remains in the interval [45,000 49,000].

This example shows that the UAR strategy to obtain the ICs around each artefact (with an ICA_P portion) seems more effective. It also preserves most of the original information between artefacts. On the other hand, UAR needs more processing and only filters the artefacts that have been previously detected.

### 5.3. Filtering an AEEG Using SASICA

**Figure 13:**
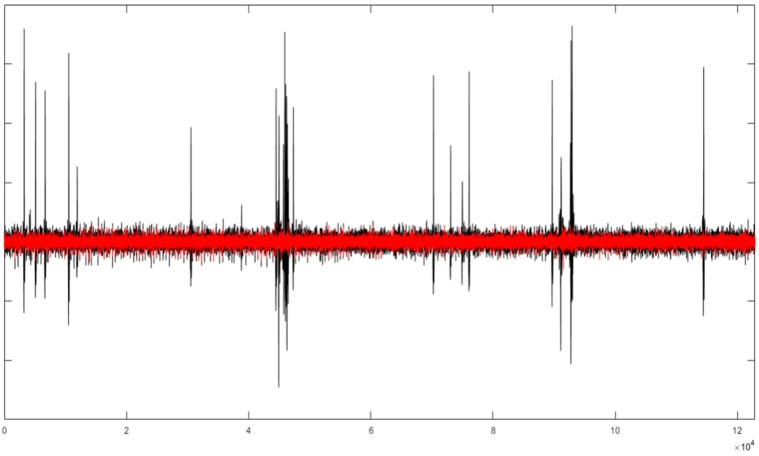
The AF8 channel of AEEG with (in black) and without artefacts (in red) after using SASICA. The *X* axis shows the samples.

The EEGLAB also has the SASICA extension [18]. The following parameters were used: Autocorrelation, Focal Components, Correlation with other channels and ADJUST selection. These parameters allow selecting the ICs related to the artefact. Figure 13 shows the AF8 channel of AEEG after using the SASICA method (in red). It is noted that SASICA selects more ICs than necessary and, consequently, there is a loss of information after removing the components. To achieve better filtering, it is necessary to analyse the selected ICs. Then, each component is accepted or rejected; but this is no longer a generic and automatic process.

### 5.4. Filtering an AEEG Using wICA

**Figure 14:**
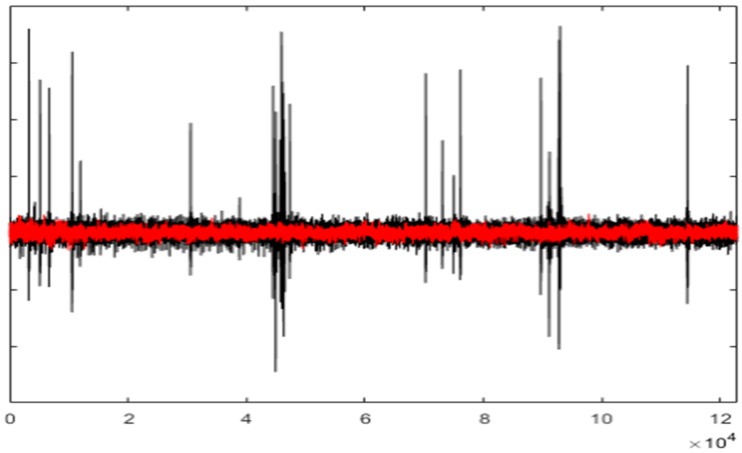
The AF8 channel of AEEG with (in black) and without artefacts (in red) after using wICA. The *X* axis shows the samples.

The AEEG has also been filtered using the wICA method [19]. This method uses stationary wavelets to enhance independent components analysis artefact removal. The following parameters have been used: a level set for stationary wavelet transform equal to 5, a threshold multiplier equal to 1 and “coif5” as wavelet family. Figure 14 shows the AF8 channel of AEEG after using the wICA method (in red). In this example wICA filters more than necessary and, consequently, there is a lost of information. Another choice of the parameters could achieve better filtering. However if this happens this method is no longer a generic and automatic process.

### 5.5. Filtering a SEEG Using UAR

**Figure 15:**
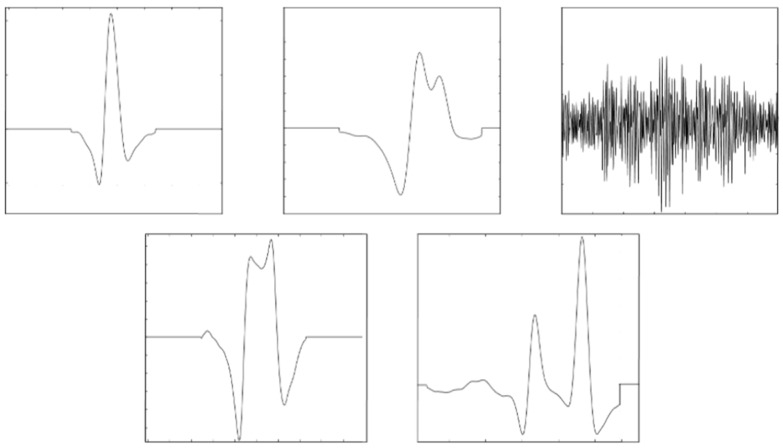
The simulated artefacts.

In this section, we filter the artefacts of a SEEG. The SEEG has 64 channels, a sampling frequency of 256 Hz and duration of 1 min. The simulated artefacts represent some eyes and EMG artefacts. The eyes artefacts have been obtained using a moving average filter over some real noisy eyes artefacts. Muscle artefacts are obtained by band-pass filtering random noise between 20 and 60 Hz. These artefacts have been projected over the channels trying to imitate the behaviour of some real artefacts. Figure 15 shows the simulated artefacts.

**Figure 16:**
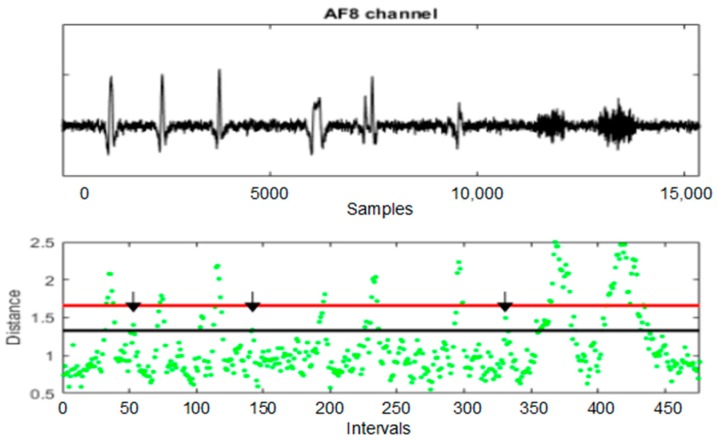
A SEEG channel, the distances of the intervals to their centroid and the thresholds *T*_1_ (in black) and *T*_2_ (in red). The black arrows point at some intervals not related to the simulated artefacts.

Figure 16 shows a channel of the SEEG and the distances of the SEEG intervals to their centroid for AD_UAR. The centroid has been calculated using all the intervals. The sliding window size is *L_w_* = 155 and the sliding distance is *d* = 32. The potential portions of signal containing artefacts appear when selecting consecutive intervals with a distance greater or equal to *T*_1_ = 1.33. The artefacts are clearly selected.

Before applying UAR to SEEG, an EEG portion without artefacts of 30 s was added at the beginning of SEEG. In this way, UAR can filter the artefacts near the beginning of SEEG.

**Figure 17:**
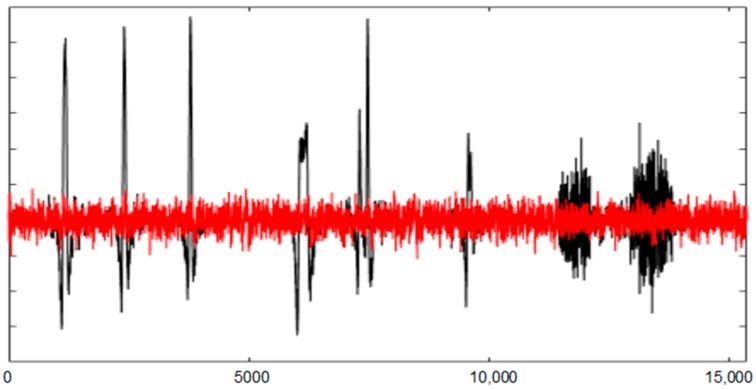
The AF8 channel of SEEG with (in black) and without artefacts (in red) after using UAR. The *X* axis shows the samples.

Figure 17 shows the entire AF8 channel of SEEG without and with filtering. Following the ordered ICs for each artefact, UAR obtains results that approximate REEG. The statistical criterion of UAR stops the filtering algorithm. Figure 16 shows some examples in which UAR has successfully removed different artefacts. Artefacts used in SEEG are in black, the corresponding portions of REEG in green and the result of the filtering using UAR in red. The FastICA algorithm has used an ICA_P of 26 s.

Differences between the filtered portions and their corresponding REEG portions are evaluated by means of the Mean Square Error (*MSE*):

(2) $$MSE=\frac{\sum_{n=1}^{M}{\left[FP_{EEG}-RP_{EEG}\right]}^{2}}{M}$$

where *FP_EEG_* is a filtered portion of *M* samples and *RP_EEG_* is its corresponding REEG portion.

**Figure 18:**
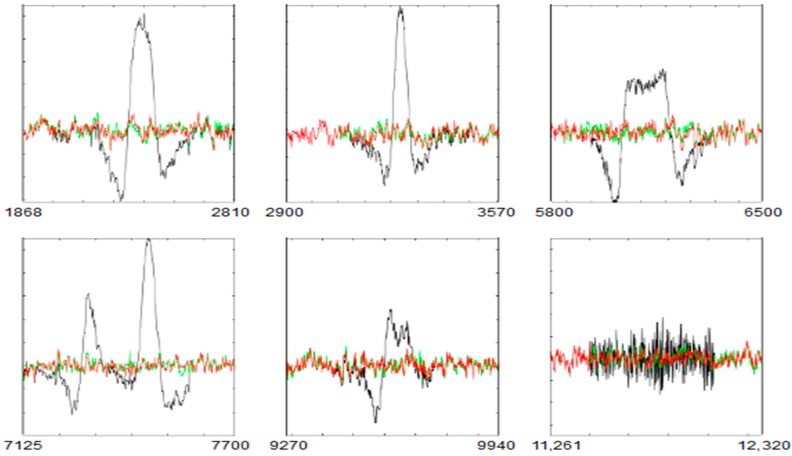
Some portions of SEEG with artefacts of the AF8 channel (in black), the corresponding *REEG* portions (in green) and the result of the filtering after using UAR (in red).

In Figure 18, following the order from left to right, the *MSE*s between the filtered portions and their corresponding parts of REEG are 7.6, 5.6 and 10.1 for the first row and 7.4, 4.7 and 5.9 for the second row.

**Figure 19:**
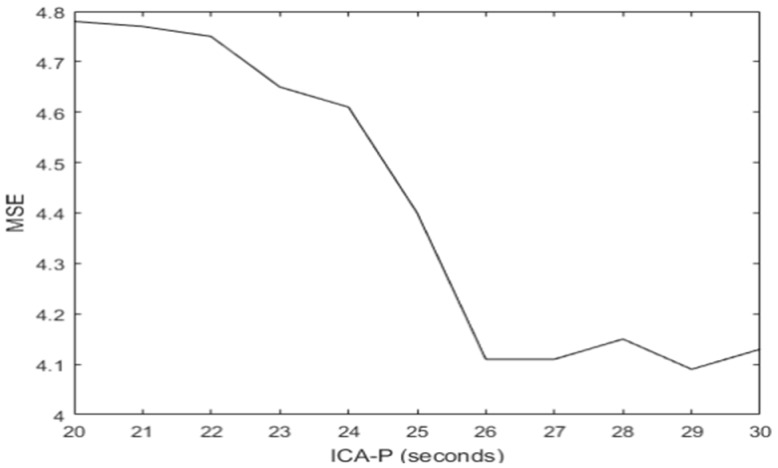
The mean squared errors (*MSE*s) (for the entire *AF*8 channel) between the filtering results and REEG from different sizes of *ICA_P*.

The quality of the UAR result depends on the quality of the ICs which in turn depends on the size of ICA_P where FastICA is applied. The SEEG allows the testing of different sizes for ICA_P. For each size, the MSE between the result of UAR over the entire SEEG and REEG (using the AF8 channel) has been calculated. From a size of ICA_P of 20 s the quality of the filtering for most of the artefacts is acceptable. However, the best result has been reached within the interval [25,30] seconds where the MSE between the filtering results and REEG are low and steady. Figure 19 shows the MSE (for the entire AF8 channel) between the filtering results and REEG from different sizes of ICA_P.

This study justifies the selection of ICA_P = 26 s that has been previously used in UAR. With this selection, the filtering is very similar to REEG. In Figure 14, the potential artefacts are portions of consecutive intervals with a distance greater or equal to *T*_1_ = 1.33. Some intervals between *T*_1_ and *T*_2_, not related to the simulated artefacts, are also selected; for example, the intervals 53, 142 and 331 (see the black arrows). The UAR method tries to filter them but the statistical criterion of UAR stops the algorithm at the beginning. Consequently, UAR only filters the simulated artefacts of SEEG.

### 5.6. Filtering a SEEG Signal Using MARA

**Figure 20:**
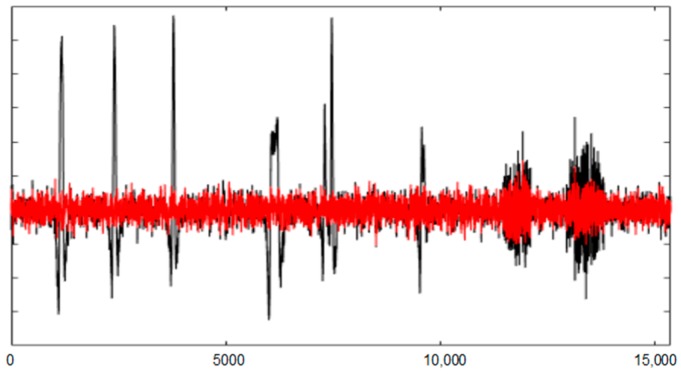
The AF8 channel of SEEG with (in black) and without artefacts (in red) after using MARA. The *X* axis shows the samples.

The SEEG signal has been filtered using MARA. Figure 20 shows the AF8 channel of SEEG after using MARA (in red). In this example, MARA has behaviour similar to the one shown in Section 5.2. MARA does not select all the necessary ICs to filter the artefacts and, consequently, the filtering is not complete; especially the EMG filtering. The *MSE* between the filtering result and REEG is 13.42, which is bigger than UAR where *MSE* = 4.1.

### 5.7. Filtering a SEEG Signal Using SASICA

**Figure 21:**
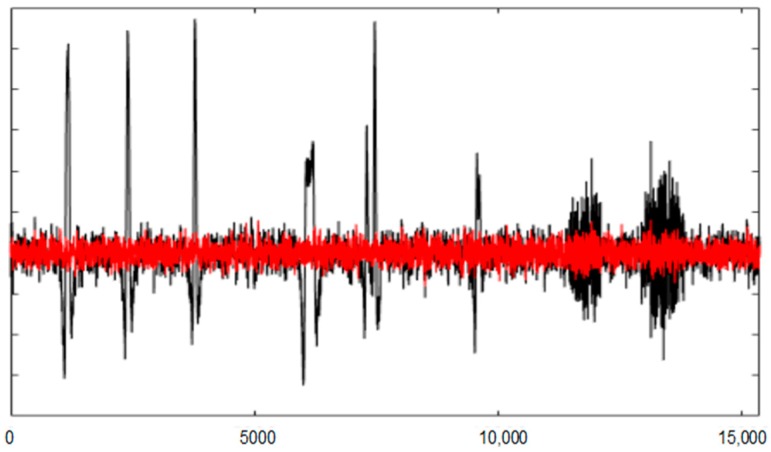
The AF8 channel of SEEG with (in black) and without artefacts (in red) after using SASICA. The *X* axis shows the samples.

The SEEG signal has been filtered using SASICA. Figure 21 shows the AF8 channel of SEEG after using SASICA (in red). In this example, SASICA has used the same parameters than Section 5.3.

The filtering has a better behaviour than the one shown in Section 5.3. However, the *MSE* between the filtering result and REEG is 16.1. Bigger than MARA and UAR.

### 5.8. Filtering a SEEG Signal Using wICA

**Figure 22:**
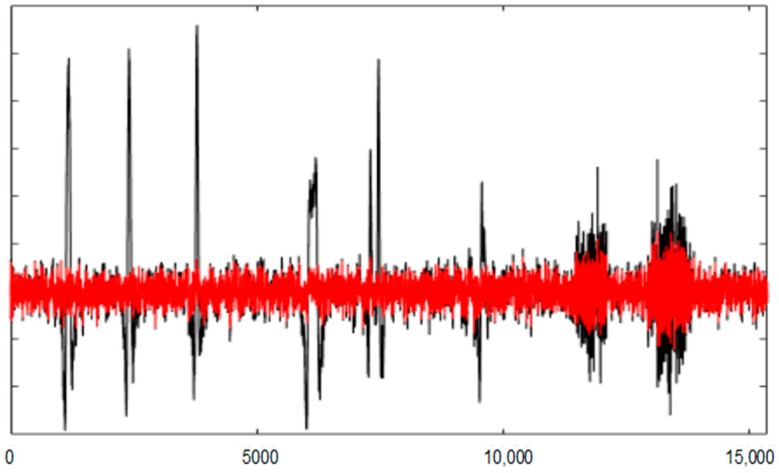
The AF8 channel of SEEG with (in black) and without artefacts (in red) after using wICA. The *X* axis shows the samples.

The SEEG signal has been filtered using wICA. Figure 22 shows the AF8 channel of SEEG after using wICA (in red). In this example, wICA has used the same parameters than Section 5.4.

The filtering has a different behaviour than the one shown in Section 5.4. In this case, the filtering is not complete; especially the EMG filtering. The MSE between the filtering result and REEG is 19.67; bigger than SASICA, MARA and UAR.

## 6. Discussion

The UAR method allows automated filtering of artefacts. This method combines unsupervised artefact detection, FastICA and unsupervised ICs selection. Each artefact detected with AD_UAR is filtered individually when the previous artefacts have been removed. Most of the original information between artefacts is preserved.

The focus of the present study is on physiological artefacts. Although UAR could also be used to filter nonphysiological artefacts, they usually are avoided through an appropriate filtering as a previous step to UAR. For example, an interference of the electrical network can be corrected by means of a band-stop filter with a narrow stopband (notch filter). The AEEG in Section 5 has been previously filtered using a notch filter to remove potential 60 Hz interferences.

In an online application, UAR_AD obtains the AR of the intervals as the AEEG is acquired. When it obtains a set of consecutive intervals with a distance to the centroid greater than T_1_, it detects a potential artefact. Then, a ICA_P of 26 s is selected. It is convenient to add a portion of EEG without artefacts before the starting point of the acquisition. In this way, it is possible the selection of ICA_P for an artefact near the starting point.

In an online application, it is sufficient to use a processor where the filtering processing time of an artefact with UAR is less than the acquisition of 2 × *d* samples; this results from the fact that after an artefact there is always at least one interval without artefact. In the most stringent case, we find after the artefact two intervals where the second is a potential artefact. While filtering occurs, these 2 × *d* samples must be saved. This is possible using ping-pong storage.

The AD_UAR is the result of a simplification of UMED. AD_UAR has been designed to operate in an unsupervised way with UAR. In AD_UAR, an optimal threshold from which the intervals with artefacts appear is not defined. In our work, we select a minimum threshold T_1_ = 1.33 (the start of the right tail) for our distribution of 64 electrodes. This implies that the selected portions of the signal using T_1_ may or may not have an artefact. If UAR does not filter a portion, that portion does not present artefacts. AD_UAR detects potential signal portions to be filtered and it is UAR which filters or not (following a statistical criterion) such portions.

The AD_UAR is a detection method to work with UAR in an unsupervised way and it does not make sense to compare it with other detection methods since it does not work with an optimal threshold. This method proposes possible portions with artefacts to UAR and the statistical criterion of UAR decides whether it is necessary to filter or not. That is, “true detection” occurs once UAR has worked on a portion selected by AD_UAR. This implies more processing, but has the advantage that AD_UAR and UAR can work in an unsupervised way and in online applications.

AD_UAR takes advantage of the AR coefficients, since, on the one hand, they are useful to detect artefacts in a simple way and, on the other, they allow us to generalize the results. These characteristics also justify why this method has been used in UAR.

In Figure 8, a small artefact not detected to be filtered is shown. This artefact has not interfered with the filtering of others artefacts. When the number of electrodes is high, that kind of artefact should not affect the global information collected from all the channels. In this case, it is not necessary to filter it. However, the AD_UAR method can improve its performance by working with a small number of electrodes or with each electrode individually. Consequently, for better detection, instead of using AD_UAR on all the channels, it can be applied to small groups of electrodes and then merge the potential artefacts detected. When the AD_UAR works with fewer channels, the Euclidean distance is more sensitive to small changes.

In theory, any ICA-based method can filter any type of artefact as long as it works with the right number of electrodes. Consequently, UAR can also correctly filter any type of artefact.

The filtering quality of a particular method varies according to the conditions of use. The quality of a method can change if, for example, the number of electrodes, the type of artefact, the selected ICA method, the internal parameters of each method, etc. also change.

In general, when working with a number of ICs less than the optimal (e.g., ICs from a portion of EEG signal that has an artefact) there is a possibility that some ICs related to the artefact have some cognitive activity. Therefore, it is important to work with a high number of electrodes and, in this way, the possible cognitive influence in the ICs related to the artefacts will be very small or nonexistent. From there, it only remains to select those ICs related to the artefacts to perform the filtering. We use a statistical criterion to obtain a good result selecting the ICs related to the artefact. We cannot quantify if some ICs related to the artefact have any cognitive influence. However, we can affirm that with a high number of electrodes, that cognitive influence will be nonexistent or very small.

UAR always works in the same way for each type of artefact. ICA is used to obtain the ICs for each artefact, (in our case using FastICA to obtain 64 ICs). Regardless of the content of the ICs, the only difference between artefacts is the number of ICs that represent each artefact. For example, a wink can be related to a small number of ICs. However, for an EMG artefact or a mixture or succession of artefacts with EMG, the number of related ICs increases significantly.

UAR sorts the ICs and uses their projections, one by one, to filter the selected artefact. Using a statistical criterion, the filtering process is stopped. This criterion (a two-sample F-Test working at 1% significance) is also a way of quantitatively assessing the quality of the result establishing the same filtering objective for any type of artefact. In UAR, this process is the same for any artefact and is independent of the type of artefact. UAR does not care about the type of artefact, it only considers a set of ICs to work with. All these reasons clearly justify why the evaluation of our filtering method does not require testing all the existing artefacts. We believe that UAR is well explained with the artefacts found in the real and simulated recordings used in this paper. More types of artefacts would not add additional information about how our methodology works.

Neither UAR nor AD_UAR depend on the origin of the test data. Consequently, the filtering process is a subject-independent algorithm. In this way, the AEEG used in this paper to test the algorithm is sufficient to analyse and evaluate it.

It is understood that UAR is only for artefacts that are mixed to the EEG signal. This method is not valid for artefacts where there is nothing to filter; for example, a disconnected electrode.

The AEEG has allowed us to show how UAR filters some real artefacts. In other EEG recordings, more types of artefacts exist. As explained above, this fact is not a problem for UAR because its filtering process does not depend on the type of artefact. When, in an AEEG, there is a large portion contaminated by artefacts, UAR filtering may not be so effective. The UAR needs a *REF* to filter an artefact. If the entire signal is contaminated, it would not be possible to select such *REF* and, therefore, UAR could not be used. However, if it is possible to select a *REF* before the large artefact, then it would be preferable to divide the artefact into segments and apply UAR sequentially.

Our method has the following characteristics.

- It uses an unsupervised detection method to detect potential signal portions of artefacts.
- The filtering process is unsupervised (it does not depend on a learning process and automatically selects ICs using a statistical criterion).
- Its strategy to obtain the ICs around each artefact (with an ICA_P portion) improves the quality of the ICs and facilitates its ordering and selection. Most of the original information between artefacts is preserved.
- It is easy to implement and can be used in online applications.
- It can be used with different types of artefacts including a portion of consecutive artefacts and any mixture of artefacts.
- To obtain good results it is necessary to work with a significant number of electrodes (e.g., 64).

The main characteristic is the automatic unsupervised selection of the ICs. It is not necessary to make a manual selection or a learning process (for an automatic selection using a machine learning algorithm).

UAR results can be evaluated by visual inspections of Figure 7, Figure 8 and Figure 9 of an AEEG and Figure 15 and Figure 16 of a SEEG. MSE has also been used to evaluate the filtering of SEEG.

The quality of the proposed UAR filtering is at least as good as that of other methods. We have compared UAR with MARA. Both are ICA-based methods and pursue the same goal: to automatically select ICs. MARA needs less processing but it is a supervised method and the quality of the ICs selection depends on the quality of a learning process. On the other hand, UAR is an unsupervised method and the ICs selection quality depends on a statistical criterion. Both methods have been tested using an AEEG and the results can be visually analysed. In general, UAR provides a better quality result than MARA. Graphics and MSE for the SEEG filtering show this fact.

We have also compared SASICA and UAR. In [18], different methods to select artefactual ICs are evaluated. They show that “their selection was not perfectly consistent, showing that there are inherent limitations to the precision of artefact selection using ICA” and, consequently, “no automated method can accurately isolate artefacts without supervision”. Some of those methods work only with some specific type of artefact. Unlike UAR, in general, SASICA does not automatically select the correct ICs to filter artefacts and needs supervision.

With the wICA method, the worst results have been obtained. In theory, the quality of the wICA filtering depends on the choice of certain parameters. Therefore, if we use the same parameters for any signal, the quality of the result may vary. If the parameters need to be changed to improve the quality, then, unlike UAR, wICA cannot be considered as a generic and automatic method.

An ICA-based method used to filter artefacts begins with the detection of the artefacts (this stage is not always done), the ICs are obtained and selected for filtering and finally the quality of the result can be analysed through a visual inspection or a quantitative evaluation. These three processes are independent. However, in our work, these stages are related to each other by the statistical criterion. The statistical criterion determines if there is filtering or not (that is, whether there is an artefact or not), it controls the filtering of the ICs projections since it decides when the process stops and, finally, it is also a way to evaluate the result, establishing the same objective for any type of artefact. Instead of using an index after filtering, the statistical criterion is itself an indirect form of quantitative evaluation of the result.

The performance of the statistical criterion to select ICs has been evaluated quantitatively with the help of a SEEG and the MSE metric. The MSE shows the difference between the filtering result using UAR for each artefact and its corresponding part of REEG. The good filtering quality of the different analysed artefacts shows that UAR correctly selects the ICs in an unsupervised way. However, the quality of the result also depends on how the ICs have been obtained. The size of ICA_P is important for ICs to capture the characteristics of the artefacts.

All UAR steps have been selected to use UAR in online applications. However, for off-line applications, UAR could benefit from data reduction techniques, such as PCA [43]. These would be applied to the vectors of autoregressive coefficients that represent the intervals obtaining new vectors. In theory, with these new vectors, AD_UAR would improve the quality of artefact detection.

ICA-based methods are well-accepted in the literature for filtering artefacts and it is not the objective of our article to evaluate their performance in relation to other methods. ICA-based methods prevail in the literature [1,44], and have proved to be satisfactory in most scenarios of interest. For example, Ref. [44] presents a comparison of artefact removal algorithms including ICA-based methods. Consequently, UAR is important insofar as it addresses the problem of automatic selection of ICs in ICA-based methods.

The UAR strategy to obtain the ICs around each artefact (with an ICA_P portion) improves the quality of the ICs and facilitates its selection. It also allows UAR to preserve most of the original information between artefacts. Figure 6 shows the ICA_P portion used to determine the ICs. However, the filtering of the ICs projections is only carried out on the area of the artefact (*AT*_1_ + *AT* + *AT*_2_). This means that the portion between the origin of ICA_P and the origin of *AT*_1_ is not altered at all. Depending on the detection of the artefact and the selection of *AT*_1_ (or *AT*_2_), the filtering may slightly alter a small portion called *EA*_1_ (or *EA*_2_), (see Figure 5) unrelated to the artefact. Unlike other methods that do not perform detection and filter using *IC*s throughout the signal, our algorithm retains most of the original information among the filtered artefacts.

A two-sample *F*-Test for equal variances is used to select the ICs related to the artefact. Other statistical tests or a combination of them can also be used. For example, the two-sample Kolmogorow-Smirnov test [45]. This test analyses if two sets of data *X*1 and *X*2 are from the same continuous distribution. It tests the null hypothesis that data in *X*1 and *X*2 come from populations with the same distribution. However, the two-sample *F*-Test has given good results working in UAR. It is especially effective when different consecutive artefacts appear.

The common issue among the ICA-based methods is the algorithm to get the ICs. It is also the part of the algorithm that needs more time. The processing time difference between UAR and other possible methods can be estimated. The processing time of an ICA algorithm depends on the window size used. Suppose *T_D_* denotes the processing time of an ICA over a window of *D* seconds. For an AEEG with *NP* artefacts, UAR will work with *MP* portions of potential artefacts with *MP* >=*NP*. If we compare UAR with another algorithm that applies ICA in the whole signal of *DT* seconds, then the processing time difference is approximately (*MP* × *T*_26_) − *T_DT_*.

In an online application, artefact detection depends on the number of electrodes. To detect a small artefact that is present in a few electrodes, we suggest to work with groups of electrodes smaller than 64. This increases the sensitivity of the detection. The ideal would be to work with a number of electrodes such that the detection of all the artefacts is coincident with the opinion of an expert. As a future task, a clinical study would allow us to study different groups of electrodes and choose the optimal group (or the optimal groups) so that the detection of the artefacts is more accurate.

## 7. Conclusions

The presented UAR method is an automatic, reliable tool developed to remove artefacts in an unsupervised way. It combines an unsupervised artefact detection method with an automatic selection of ICs after using the FastICA algorithm for each artefact individually. This method can be used in online applications.

The two-sample *F*-test used to select the ICs also offers a statistical criterion to justify the filtering process. Furthermore, the results of the algorithm can be visually analysed.

The UAR filtering on EEG test signals with different types of artefacts has been correct. This means that the unsupervised strategy proposed for selecting the ICs works. As UAR is unsupervised, it should be able to filter any artefact, regardless on their number or type.

## References

[1] Urigüen J.A., Garcia-Zapirain B. (2015). EEG artefact removal—State-of-the-art and guidelines. J. Neural Eng..

[2] Nolan H., Whelan R., Reilly R.B. (2010). FASTER: Fully Automated Statistical Thresholding for EEG artefact Rejection. J. Neurosci. Methods.

[3] Sörnmo L., Laguna P. (2005). Bioelectrical Signal Processing in Cardiac and Neurological Applications.

[4] Lance B.J., Kerick S.E., Ries A.J., Oie K.S., McDowell K. (2012). Brain-computer interface technologies in the coming decades. Proc. IEEE.

[5] James C.J., Gibson O.J. (2003). Temporally constrained ICA: An application to artefact rejection in electromagnetic brain signal analysis. IEEE Trans. Biomed. Eng..

[6] Pham T.T.H., Croft R.J., Cadusch P.J., Barry R.J. (2011). A test of four EOG correction methods using an improved validation technique. Int. J. Psychophysiol..

[7] Croft R.J., Barry R.J. (2000). Removal of ocular artefact from the EEG: A review. Neurophysiol. Clin..

[8] Sweeney K.T., Ward T.E., McLoone S.F. (2012). Artefact removal in physiological signals-practices and possibilities. IEEE Trans. Inf. Technol. Biomed..

[9] James C.J., Hesse C.W. (2005). Independent component analysis for biomedical signals. Physiol. Meas..

[10] Vigário R., Oja E. (2008). BSS and ICA in Neuroinformatics: From Current Practices to Open Challenges. IEEE Rev. Biomed. Eng..

[11] Jung T.P., Makeig S., Humphries C., Lee T.W., McKeown M.J., Iragui V., Sejnowski T.J. (2000). Removing electroencephalographic artefacts by blind source separation. Psychophysiology.

[12] Junghöfer M., Elbert T., Tucker D.M., Rockstroh B. (2000). Statistical control of artefacts in dense array EEG/MEG studies. Psychophysiology.

[13] Oostenveld R., Fries P., Maris E., Schoffelen J.-M. (2011). FieldTrip: Open source software for advanced analysis of MEG, EEG, and invasive electrophysiological data. Comput. Intell. Neurosci..

[14] Delorme A., Makeig S. (2004). EEGLAB: An open source toolbox for analysis of single-trial EEG dynamics including independent component analysis. J. Neurosci. Methods.

[15] Lawhern V., Hairston W.D., Robbins K. (2013). DETECT: A MATLAB Toolbox for Event Detection and Identification in Time Series, with Applications to artefact Detection in EEG Signals. PLoS One.

[16] Mur A., Dormido R., Vega J., Duro N., Dormido-Canto S. (2016). Unsupervised event characterization and detection in multichannel signals: An EEG application. Sensors.

[17] Winkler I., Haufe S., Tangermann M. (2011). Automatic Classification of artefactual ICA-Components for artefact Removal in EEG Signals. Behav. Brain Funct..

[18] Chaumon M., Bishop D.V., Busch N.A. (2015). A practical guide to the selection of independent components of the electroencephalogram for artifact correction. J. Neurosci. Methods.

[19] Castellanos N.P., Makarov V.A. (2006). Recovering EEG brain signals: Artifact suppression with wavelet enhanced independent component analysis. J. Neurosci. Methods.

[20] Melia U., Clariá F., Vallverdú M., Caminal P. (2014). Filtering and thresholding the analytic signal envelope in order to improve peak and spike noise reduction in EEG signals. Med. Eng. Phys..

[21] Mahajan R., Morshed B. (2014). Unsupervised Eye Blink artefact Denoising of EEG Data with Modified Multiscale Sample Entropy, Kurtosis and Wavelet-ICA. IEEE J. Biomed. Heal. Informatics..

[22] Daly I., Scherer R., Billinger M., Muller-Putz G. (2014). FORCe: Fully online and automated artefact Removal for brain-Computer interfacing. IEEE Trans. Neural Syst. Rehabil. Eng..

[23] Zeng K., Chen D., Ouyang G., Wang L., Liu X., Li X. (2015). An EEMD-ICA Approach to Enhancing artefact Rejection for Noisy Multivariate Neural Data. IEEE Trans. Neural Syst. Rehabil. Eng..

[24] Chen X., Liu A., Chen Q., Liu Y., Zou L., McKeown M.J. (2017). Simultaneous ocular and muscle artifact removal from EEG data by exploiting diverse statistics. Comput. Biol. Med..

[25] Somers B., Francart T., Bertrand A. (2018). A generic EEG artifact removal algorithm based on the multi-channel Wiener filter. J. Neural Eng..

[26] Mannan M.M.N., Kamran M.A., Jeong M.Y. (2018). Identification and Removal of Physiological Artifacts from Electroencephalogram Signals: A Review. IEEE Access.

[27] Islam M.K., Rastegarnia A., Yang Z. (2016). Methods for artifact detection and removal from scalp EEG: A review. Neurophysiol. Clin./Clin. Neurophysiol..

[28] Chen X., Liu A., Chiang J., Wang Z.J., McKeown M.J., Ward R.K. (2016). Removing Muscle Artifacts from EEG Data: Multichannel or Single-Channel Techniques?. IEEE Sens. J..

[29] Burg J.P. Maximum Entropy Spectral Analysis. Proceedings of the 37th Annual International SEG Meeting.

[30] Rokach L., Maimon O. (2005). Chapter 15—Clustering Methods.

[31] Halkidi M., Vazirgiannis M. Clustering validity assessment: Finding the optimal partitioning of data set. Proceedings of the 2001 IEEE International Conference on Data Mining.

[32] Lawhern V., Hairston W.D., McDowell K., Westerfield M., Robbins K. (2012). Detection and classification of subject-generated artifacts in EEG signals using autoregressive models. J. Neurosci. Methods.

[33] Mur A., Dormido R., Vega J., Dormido-Canto S., Duro N. (2016). Unsupervised event detection and classification of multichannel signals. Expert Syst. Appl..

[34] Wang G., Ding Q., Hou Z. (2008). Independent component analysis and its applications in signal processing for analytical chemistry. Trends Anal. Chem..

[35] Hyvärinen A., Oja E. (1997). A Fast Fixed-Point Algorithm for Independent Component Analysis. Neural Comput..

[36] Cardoso J.F., Souloumiac A. (1993). Blind beamforming for non-gaussian signals. IEE Proceedings F (Radar and Signal Processing).

[37] Bell A.J., Sejnowski T.J. (1995). An information-maximization approach to blind separation and blind deconvolution. Neural Comput..

[38] Astakhov S.A., Stögbauer H., Kraskov A., Grassberger P. (2006). Monte Carlo algorithm for least dependent non-negative mixture decomposition. Anal. Chem..

[39] Learned-Miller E.G., Fisher III J.W. (2003). ICA Using Spacings Estimates of Entropy. J. Mach. Learn. Res..

[40] Delorme A., Palmer J., Onton J., Oostenveld R., Makeig S. (2012). Independent EEG sources are dipolar. PLoS ONE.

[41] Albera L., Kachenoura A., Comon P., Karfoul A., Wendling F., Senhadji L., Merlet I. (2012). ICA-based EEG denoising: A comparative analysis of fifteen methods. Bull. Polish Acad. Sci. Tech. Sci..

[42] Snedecor G.W., Cochran W.G. (1989). Statistical Methods.

[43] Jolliffe I.T. (2002). Principal Component Analysis, Second Edition. Encycl. Stat. Behav. Sci..

[44] Urigüen J.A., Garcia-Zapirain B. (2017). Validation EEG artefact removal. J. Med. Imag. Health Inform..

[45] Massey M.J. (1951). The Kolmogorov-Smirnov Test for Goodness of Fit. J. Am. Stat. Assoc..

